# Plasmid genomic epidemiology of blaNDM carbapenemase-producing Enterobacterales in Canada from 2010 to 2023

**DOI:** 10.1099/mgen.0.001415

**Published:** 2025-08-04

**Authors:** Nicole Lerminiaux, Ken Fakharuddin, Yves Longtin, Erin McGill, Robyn Mitchell, Laura Mataseje, On behalf of the Canadian Nosocomial Infection Surveillance Program

**Affiliations:** 1 National Microbiology Laboratory, Public Health Agency of Canada, Winnipeg, Manitoba, Canada; 2 Jewish General Hospital, Montréal, Québec, Canada; 3 Public Health Agency of Canada, Ottawa, Ontario, Canada

**Keywords:** *β*-lactamase, antimicrobial resistance, carbapenemase, epidemiology, genomics, plasmid, surveillance

## Abstract

Carbapenems are broad-spectrum antibiotics that are losing effectiveness against infections caused by multidrug-resistant *Enterobacterales* that have acquired carbapenemase genes. The New Delhi metallo-*β*-lactamase (*bla*_NDM_) is one of the most common carbapenemases in Canada and around the globe. These genes are frequently found on conjugative plasmids, which can disseminate through horizontal gene transfer. We applied whole-genome sequencing to characterize 1,032 *bla*_NDM_ carbapenemase-producing *Enterobacterales* isolates collected by the Canadian Nosocomial Infection Surveillance Program from 2010 to 2023. Using a combination of short-read and long-read sequencing, we obtained 226 complete and circular *bla*_NDM_-encoding plasmids. Unlike other carbapenemases in Canada, we found that *bla*_NDM_ plasmids were very diverse; there was a lack of dominant clusters identified using MOB-suite, and clustering methods were not able to accurately predict plasmid clusters for short-read-only data. The majority of *bla*_NDM_ plasmids were IncF-type (69.0%, 156/226). Both *bla*_NDM_ and *bla*_OXA-48-type_ carbapenemase genes were found in 11.4% (118/1,032) of isolates, and we identified several instances of both carbapenemase genes co-harboured on the same plasmid replicon (*n*=9). Our findings highlight that plasmid transfer has not played a major role in *bla*_NDM_ transmission across Canada and that long-read sequencing is essential for resolving *bla*_NDM_ plasmid structure and cluster membership.

**Impact Statement**

Resistance to critically important carbapenem antibiotics is a global public health threat. The dissemination of carbapenemase resistance genes is significantly facilitated by plasmids, which are mobile genetic elements that can transfer between unrelated species and strains. Consequently, understanding the features and distribution of carbapenemase-encoding plasmids is crucial for pathogen surveillance and mitigation of resistance. In this work, we used long-read and short-read sequencing to characterize the genomic epidemiology of NDM carbapenemase-encoding plasmids across 14 years of *Enterobacterales* surveillance data in Canada. Examining the genomic context of NDM carbapenemases revealed the diversity in the plasmid reservoir in Canada, which enhances future international pathogen surveillance.

## Data Summary

Raw sequencing reads were deposited to the NCBI SRA archive under BioProject PRJNA855907 (https://www.ncbi.nlm.nih.gov/bioproject/?term=PRJNA855907); see Table S1 for a list of accessions. Complete *bla*_NDM_ and *bla*_OXA-48-type_-encoding plasmid sequences were deposited to NCBI GenBank under the accessions listed in Table S2.

## INTRODUCTION

Carbapenems are critically important antimicrobials for the treatment of severe infections caused by multidrug-resistant Gram-negative bacteria. Following the introduction of carbapenem antibiotics, carbapenem-resistant pathogens in the *Enterobacterales* order have emerged through acquisition of carbapenemase genes [1]. Among Gram-negative bacteria, resistance to carbapenems increased more compared to any other antimicrobial class from 1990 to 2021 globally [2], and carbapenem-resistant *Enterobacterales* remain designated as a Critical Group on the World Health Organization Bacterial Priority Pathogen List [3] and an Urgent Threat on the Centers for Disease Control and Prevention Antibiotic Resistance Threats Report [4]. The worldwide emergence of carbapenemase-carrying bacteria poses a major challenge to public health [5].

Of the different classes of carbapenemases, New Delhi metallo-*β*-lactamase (*bla*_NDM_) is one of the most commonly identified carbapenemases in Canada [6] and around the globe [7,9]. The *bla*_NDM_ genes are a class B carbapenemase that can hydrolyse most *β*-lactams, including carbapenems [9,10]. More than 88 amino acid variants are described in NCBI’s Pathogen Detection Reference Gene Catalog to date [11]. According to the Canadian Nosocomial Infection Surveillance Program (CNISP), carbapenemase-producing *Enterobacterales* infection rates increased by 55% from 2021 to 2023 (0.09 to 0.14 per 10,000 patient days) [12]. From 2010 to 2023, *bla*_NDM_ was the second most common carbapenemase detected in Canada (29%) after *bla*_KPC_ (50%), while *bla*_NDM_ was significantly associated with international travel and receipt of medical care abroad [12].

Global expansion of *bla*_NDM_ is predicted to have peaked between 2013 and 2015 [13]. The *bla*_NDM_ genes have been found in at least 11 bacterial families and are predominantly found in *Escherichia coli* and *Klebsiella pneumoniae* from a variety of clonal lineages [8,9, 13, 14]. Common high-risk clonal lineages carrying *bla*_NDM_ are sequence types (STs) ST167, ST405, ST410, ST361 and ST648 in *E. coli*, and clonal complexes ST11 (including ST340 and ST437), ST147 and ST395 in *K. pneumoniae* [15,24].

The widespread dissemination of carbapenemase genes can be largely attributed to plasmids and transposons [13], and mobility is possible at multiple nested genetic levels [25]. Horizontal transfer of *bla*_NDM_ is mediated by multiple types of plasmids, with the most common replicon types being IncF-type, IncH-type, IncX3 or IncC [8,13, 26]. Plasmid-mediated outbreaks of *bla*_NDM_-encoding bacteria have been reported worldwide [8,27,30]. Often, plasmids harbouring *bla*_NDM_ carry additional resistance mechanisms to other classes of antimicrobials [9,26]. The Tn*125* transposon has been proposed as the ancestral transposon responsible for mobilizing *bla*_NDM_ [31], and other transposons and insertion sequences have been implicated in *bla*_NDM_ mobility and genetic reshuffling [13,22, 23, 32].

Here, we applied whole-genome sequencing to characterize the molecular epidemiology of *bla*_NDM_ carbapenemase-producing *Enterobacterales* from the CNISP from 2010 to 2023. Using a combination of short-read (Illumina) and long-read (Oxford Nanopore Technologies, ONT) sequencing of selected representatives, we investigated the diversity of complete *bla*_NDM_-encoding plasmids among these Canadian isolates and compared them to the global context of *bla*_NDM_.

## METHODS

### Surveillance period and PCR confirmation of *bla*_NDM_ carbapenemase gene

The CNISP is a sentinel surveillance system that collects epidemiological and linked microbiology data from 106 Canadian acute care hospitals across 10 provinces and 1 territory. *Enterobacterales* isolated from patients between 2010 and 2023 were eligible for inclusion by minimum inhibitory concentration above clinical breakpoints [33] or if they tested positive using molecular (PCR) or phenotypic testing (mCIM, CARBA-NP) [34]. Serial isolates from the same patient were included if the organism or carbapenemase gene differed. Further information about CNISP can be found online (https://health-infobase.canada.ca/cnisp/). Eligible isolates were submitted to the National Microbiology Laboratory (Winnipeg, Canada) for *bla*_NDM_ carbapenemase gene confirmation by multiplex PCR as previously described [35]. A total of 1,032 isolates encoding *bla*_NDM_ were collected from 2010 to 2023 from 66 hospital sites (Table S1, available in the online Supplementary Material). Where applicable, the Central region refers to the provinces of Ontario and Québec, the West region refers to the provinces of British Columbia, Alberta, Saskatchewan and Manitoba and the East region refers to the provinces of Nova Scotia, New Brunswick, Prince Edward Island and Newfoundland and Labrador.

### Species complex definitions

The organism genus was confirmed using the RefSeq Masher Matches tool v0.1.2 [36]. We used the following definitions for species complexes: the *K. pneumoniae* species complex includes *K. pneumoniae*, *Klebsiella quasipneumoniae* and *Klebsiella variicola* [37]; the *Enterobacter cloacae* complex includes *E. cloacae*, *Enterobacter hormachei*, *Enterobacter asburiae*, *Enterobacter kobei* and *Enterobacter ludwigii* [38]. To assign species, Kleborate v2.2.0 [37] was used for species identification of *Klebsiella* spp. with default parameters. Genomic clades and clusters in the *Enterobacter* spp. and *Citrobacter* spp. were defined by a pairwise average nucleotide identity-based distance matrix using FastANI v1.3 [39,42], and the clade was assigned when the mean average nucleotide identity value was >95.0%.

### Whole-genome sequencing and assembly

All 1,032 isolates were sequenced with Illumina platforms, and 237 of these were additionally sequenced using ONT. Isolates for ONT long-read sequencing represented ~23.0% (237/1,032) of all *bla*_NDM_ cases. Samples isolated prior to 2021 were selected for long-read sequencing to maintain representative proportions of each province (*n*=101), and additionally, we sequenced all isolates encoding both *bla*_NDM_ and *bla*_OXA-48-type_ from this period (*n*=54). No other factors (i.e. hospital site, specimen type and organism) were considered for selection. Based on preliminary analyses, we selected an additional 82 isolates from 2022 to 2023 for ONT sequencing from plasmid clusters that were under-represented in our dataset or that had *bla*_NDM_ contigs that did not group into existing plasmid clusters.

Genomic DNA was extracted using Epicentre MasterPure^™^ Complete kits (Mandel Scientific, Guelph, ON, Canada) for ONT from 2015 to 2021, the DNeasy 96 Blood and Tissue kit (QIAGEN, Hilden, Germany) for Illumina from 2010 to 2021 and the Mag-Bind^®^ Universal Pathogen DNA 96 kit (Omega Bio-Tek, Norcross, Georgia, USA) for Illumina and ONT from 2022 to 2023. The same DNA extract was used for both short-read Illumina and long-read ONT sequencing where possible. Short-read libraries were created with TruSeq Nano DNA HT sample preparation kits (Illumina, San Diego, CA, USA) from 2010 to 2021 or the NexteraXT library preparation kits (Illumina) from 2022 to 2023. Paired-end, 301 bp indexed reads were generated on an Illumina MiSeq^™^ platform (Illumina) from 2010 to 2021 and a NextSeq 2000 platform (Illumina) from 2022 to 2023. Long-read sequences were generated using the Rapid Barcoding Kit (SQK-RBK004) or the Rapid Barcoding Kit 96 (SQK-RBK110.96) on R9.4.1 flow cells with the MinION Mk1B (ONT, Oxford, Oxfordshire, UK). Read data were basecalled and demultiplexed with Guppy v6.5.7 using the Super High Accuracy model (ONT). Average Illumina depth of coverage was 107X, and average ONT depth of coverage was 80X.

### Bioinformatic analyses

The assembly workflows were managed using Snakemake v7.24.0 [43]. ONT reads were trimmed with Porechop v0.2.3_seqan2.1.1 [44] using default parameters and filtered for Q-score >10 and length >1,000 bases with Filtlong v0.2.1 [45] (parameters: --min_length 1000 min_mean_q 90). Illumina reads had adaptors trimmed and were filtered for an average Q-score >30 with trim-galore v0.6.7 [46] (parameters: --paired --quality 30). FastQC v0.11.9 [47] and NanoPlot v1.28.2 [48] were used to assess quality control metrics for Illumina and ONT reads, respectively. If average read depth for either Illumina or ONT reads exceeded 100X coverage, assuming a 5.2 Mb genome size, reads were randomly downsampled to 100X coverage with rasusa v2.0.0 [49]. Isolates with Illumina-only data were assembled with Unicycler v0.5.0 [50] using default settings. Those with Illumina and ONT data were first assembled with Hybracter v0.7.1 [51] (parameters: --skip_qc --medakaModel r941_min_sup_g507) (*n*=184/237). If Hybracter failed (largest chromosome contig <4 Mb, indicative of low coverage) or if the *bla*_NDM_/*bla*_OXA-48-type_ was on an incomplete/non-circular contig, then hybrid Unicycler v0.5.0 [50] was run using default settings (*n*=35/237). If the *bla*_NDM_/*bla*_OXA-48-type_ gene was on an incomplete/non-circular contig in the Unicycler assembly and if there was sufficient depth of ONT reads (>60X), a consensus assembly was generated with Trycycler v0.5.0 [52] using default parameters (*n*=18/237). For Trycycler assemblies, 12 long-read subsets with an average depth of 60X, assuming an average genome size of 5.2 Mb, were generated for each sample using the Trycycler subsample command, and 4 subsets were assembled independently with each of the following assembly tools: Flye v2.9.2 [53] (parameters: --nano-hq), Raven v1.8.1 [54] with default settings and Miniasm v0.3_r179/Minipolish v0.1.2 [55] with default settings. Trycycler assemblies were polished with Medaka v1.7.2 [56], and all assemblies were polished with short reads using Polypolish v0.5.0 [57] using default settings and POLCA from MaSuRCA v4.0.9 [58] using default settings. A total of 292 carbapenemase-encoding plasmids were circularized from these assemblies (Table S2). Of the isolates with ONT sequencing, 224/237 had *bla*_NDM_ genes on complete/circular plasmids or chromosomes.

StarAMR v0.10.0 [59] was used to detect antimicrobial resistance genes (ARGs) using the ResFinder database v2022-05-24 [60], which is a nucleotide database containing both acquired and some chromosomal/intrinsic beta-lactamases, and ST using the MLST database v2.23.0 [61,62]. All carbapenemase genes identified here had 100% nucleotide identity to the reference sequences in the ResFinder database, with two exceptions, which had 100% identity at the amino acid level. Plasmid taxonomic unit (PTU) designations were obtained from COPLA v1.0 [63]. The MOB-typer tool from MOB-suite v3.1.9 [64,65] was used to identify plasmid replicons and mobility class using the default databases. ISEscan v1.7.2.3 [66] was used to identify insertion sequence elements. Fisher’s exact test was used for differences in ARG abundance among genera, and Wilcoxon’s rank sum test was used for differences in the number of insertion elements per replicon type. Single nucleotide variants (SNV) were determined using SNVPhyl v1.2.3 [67] with the following parameters: minimum base coverage 10, minimum mapping quality score 30, relative SNV abundance ratio 0.75 and window size for SNV density filtering 20. Code for the splitting tree of transposon structural variants was adapted from Acman *et al*. [13,68].

Plots were created using R v4.4.0 [69] and the following packages: tidyverse packages [70], patchwork v1.1.2 [71], ggpubr v0.6.0 [72], ggsankey v0.0.99 [73], gggenes v0.3.0 [74], ComplexHeatmap v2.20 [75] and treemapify v2.5.6 [76]. A circular plasmid map was created with Proksee [77] by running blastn v2.12.0+ [78], Prokka v1.14.6 [79] and mobileOG-db v1.6 [80]. Plasmid alignments were created with blastn v2.16.0+ [78], Prokka v1.14.6 [79], mobileOG-db v1.6 [80], GenoFig v1.1.1 [81] and Inkscape v1.4 [82].

### Plasmid clustering and containment analysis

For plasmid clustering analysis, the PLSDB v2021_06_23v2 [83] database was downloaded and clustered alongside the 292 circular carbapenemase plasmids in this study using MOB-cluster from the MOB-suite v3.1.9 package [64,65] using default parameters. The primary and secondary cluster numbers generated here are unique to this manuscript and differ from those used in the default MOB-suite database. All 1,032 isolates were screened for plasmids with MOB-recon using the MOB-suite default database, and the output was filtered to focus on the reconstructed plasmids containing carbapenemase genes.

Pling calculates Double Cut and Join Indel (DCJ-Indel) distances between plasmids, constructs a (transposable element-aware) relatedness network and then finds clusters of closely related plasmids using a network community algorithm [84]. We used pling v2.0 with default thresholds: containment threshold of 0.5 and DCJ-Indel distance of 4.

## RESULTS

### Characteristics of *bla*_NDM_ carbapenemase-producing isolates

**Fig. 1.**
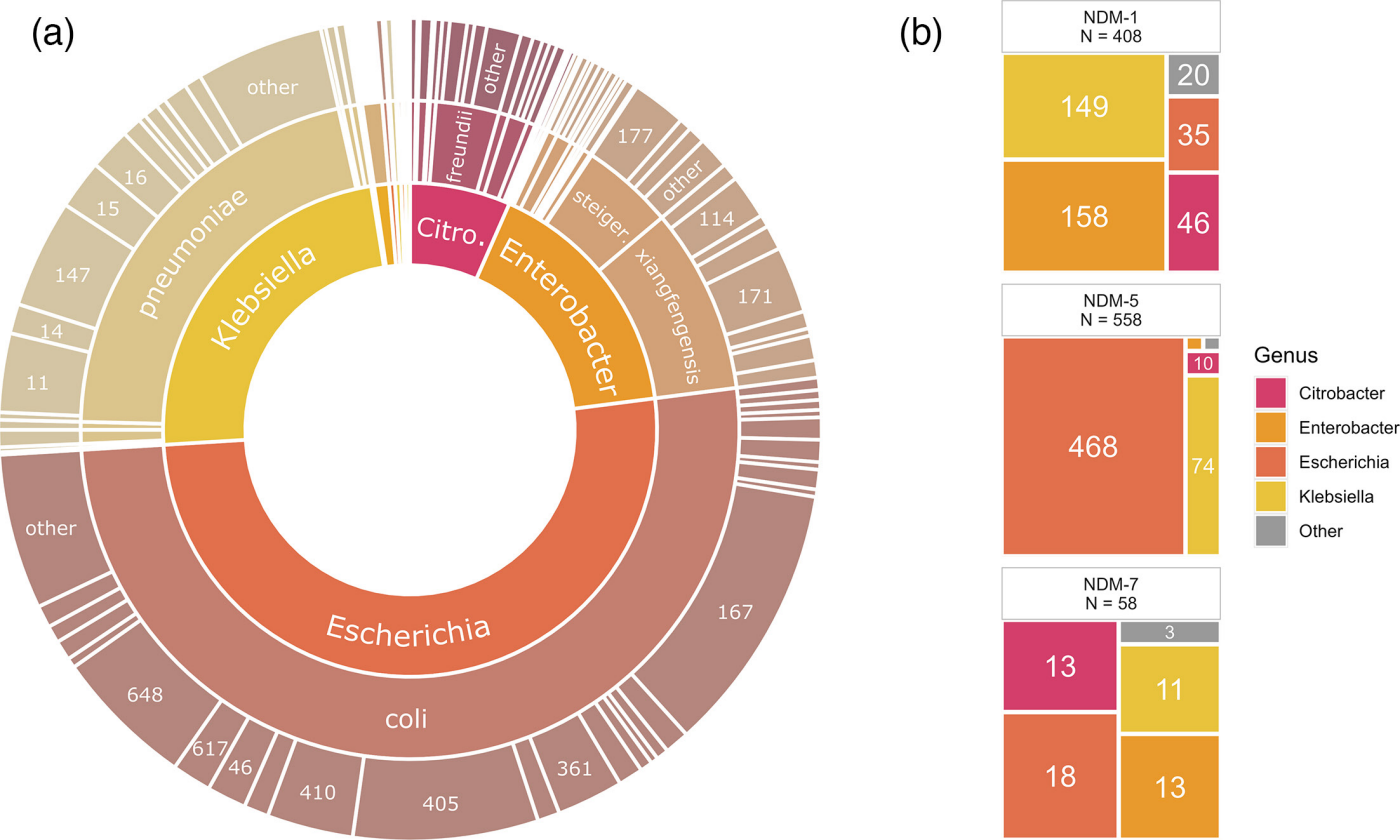
(**a**) Summary of genera (inner ring), species (middle ring) and multi-locus species types (MLST; outer ring) of *bla*_NDM_-encoding isolates included in the study (1,032 total isolates). MLST profiles found in three or fewer isolates were grouped into ‘other’. Not all labels are displayed, and colour is meaningless. ‘Citro.’ is an abbreviation for *Citrobacter*, and ‘steiger.’ is an abbreviation for subsp. *steigerwaltii*. (**b**) Count of most common *bla*_NDM_ alleles (*bla*_NDM-1_, *bla*_NDM-5_, *bla*_NDM-7_) by genus. The area of tiles corresponds to the number of isolates. Isolates with other *bla*_NDM_ types (*n*=24) were excluded. ‘Other’ genera include *Leclercia*, *Mixta*, *Morganella*, *Proteus*, *Providencia*, *Raoultella* and *Serratia* (*n*=26).

A total of 1,032 non-duplicate *bla*_NDM_-encoding isolates were submitted by 66 hospital sites across Canada from 2010 to 2023 (5 in the East region, 29 in the Central region and 32 in the West region) (Table S1). Serial isolates from the same patient were included if the organism or carbapenemase gene differed. We performed short-read sequencing on all isolates and long-read sequencing on a subset of 237 isolates, which we selected to maintain representative proportions of each province in Canada (see Methods for details). The *bla*_NDM_-producing isolates belonged to 12 genera and 32 species, with the most common species being *E. coli* (527/1,032, 51.1%), *K. pneumoniae* species complex (228/1,032, 22.1%) and *E. cloacae* species complex (162/1,032, 15.7%) (Fig. 1a). The most common STs within *E. coli* were ST167 (110/528, 20.8%), ST405 (75/528, 14.2%), ST648 (57/528, 10.8%) and ST410 (35/528, 6.7%), overall representing 52.5% of all *bla*_NDM_ *E. coli* isolates. Other common STs were *K. pneumoniae* ST147 (44/228, 19.3%), *K. pneumoniae* ST11 (32/228, 14.0%) and *Enterobacter hormaechei* ST171 (27/162, 16.7%).

**Fig. 2.**
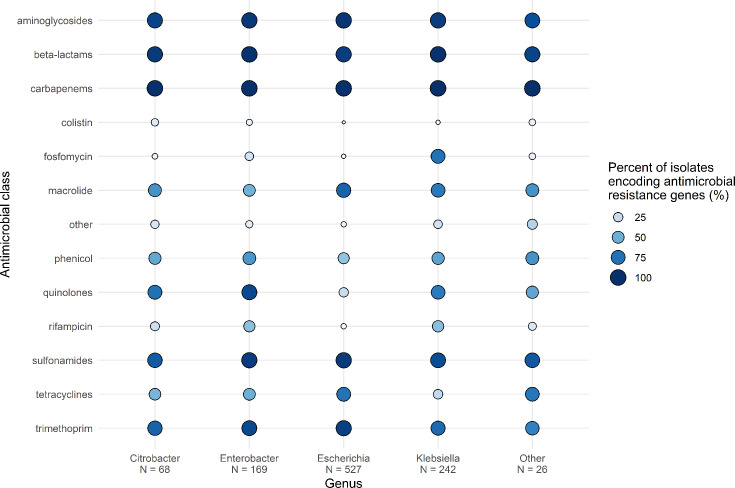
Proportion of *bla*_NDM_-encoding isolates encoding ARGs identified by StarAMR across the top four genera. Values represent the proportion of isolates that encode an ARG that provides resistance to that antimicrobial class. The ‘beta-lactams’ category does not include carbapenemase genes (*bla*_NDM_, *bla*_OXA_, *bla*_KPC_). ‘*N*’ indicates number of isolates in that genus. ‘Other’ genera include *Leclercia*, *Mixta*, *Morganella*, *Proteus*, *Providencia*, *Raoultella* and *Serratia*.

Using StarAMR for detection of resistance genes in the genome sequencing data [59], we observed 97.1% (1,002/1,032) of isolates harboured additional *β*-lactamase genes alongside the *bla*_NDM_ carbapenemase gene (Fig. 2, Table S1). Of the 1,002 isolates harbouring additional *β*-lactamases, *bla*_CTX-M-15_ (474/1,002, 47.3%), *bla*_TEM-1B_ (463/1,002, 46.2%), *bla*_OXA-1_ (420/1,002, 41.9%), *bla*_CMY-42_ (100/1,002, 10.0%), *bla*_ACT-16_ (94/1,002, 9.4%) and *bla*_OXA-10_ (91/1,002, 9.1%) were the most common types. Sulphonamide, trimethoprim, chloramphenicol and aminoglycoside resistance genes were commonly observed among multiple genera; the most common genes included *sul1* (sulphonamides, 920/1,032, 89.1%), *mphA* (macrolides, 653/1,032, 63.2%), *aadA2* (aminoglycosides, 517/1,032, 50.0%), *aac*(6′)-Ib-cr (aminoglycosides, 510/1,032, 49.4%), *dfrA12* (trimethoprim, 509/1,032, 49.3%), *catB3* (phenicols, 392/1,032, 40.0%) and *sul2* (sulphonamides, 311/1,032, 30.1%). No isolates were observed to encode the mobile tigecycline resistance gene cluster *tmexCD-toprJ*.

There were notable differences in resistance gene carriage among different genera. Colistin resistance genes were most likely to be found in *Enterobacter* spp. than *E. coli* or *Klebsiella* spp. (6.0% vs 0.2% vs 1.2%, respectively, *P*<0.001). Trimethoprim resistance genes were most likely to be found in *E. coli* or *Enterobacter* spp. than *Klebsiella* spp. (94.0% vs 90.0% vs 78.0%, respectively, *P*<0.001). Fosfomycin resistance genes were most likely to be found in *Klebsiella* spp., followed by *Enterobacter* spp. and *E. coli* (75.0% vs 19.0% vs 1.3%, respectively, *P*<0.001). Quinolone resistance genes were most likely to be found in *Enterobacter* spp., followed by *Klebsiella* spp. and *E. coli* (91.0% vs 71.0% vs 25.0%, respectively, *P*<0.001).

A total of 1,048 *bla*_NDM_ genes were detected among the 1,033 isolates, with 1.1% (11/1,032) of isolates harbouring more than one *bla*_NDM_ copy (Table S1). The *bla*_NDM-5_ variant was the most common (558/1,048, 53.2%), followed by *bla*_NDM-1_ (408/1,048, 38.9%), *bla*_NDM-7_ (58/1,048, 5.5%), *bla*_NDM-4_ (14/1,048, 1.3%) and *bla*_NDM-19_ (3/1,048, 0.3%) (Fig. 1b). Two isolates had *bla*_NDM-6_, two isolates had *bla*_NDM-39_ and three isolates encoded *bla*_NDM-3_, *bla*_NDM-76_ and *bla*_NDM-78_, respectively. There were four (4/1,032, 0.4%) isolates that co-harboured different *bla*_NDM_ alleles in the same cell (two *bla*_NDM-1_ and *bla*_NDM-4_; one *bla*_NDM-1_ and *bla*_NDM-5_; and one *bla*_NDM-1_ and *bla*_NDM-7_). Additionally, 11.4% (118/1,032) of isolates co-harboured a *bla*_OXA-48-type_ carbapenemase. There was a total of 123 *bla*_OXA-48-type_ genes detected, with the most common being *bla*_OXA-181_ (57/123, 46.3%), *bla*_OXA-232_ (43/123, 35.0%), *bla*_OXA-48_ (9/123, 7.3%), *bla*_OXA-1207_ (5/123, 4.1%), *bla*_OXA-1205_ (3/123, 2.4%), *bla*_OXA-484_ (3/123, 2.4%), *bla*_OXA-244_ (2/123, 1.6%) and *bla*_OXA-1181_ (1/123, 0.8%). Furthermore, 13 isolates encoded 14 *bla*_KPC_ carbapenemases (13/1,032, 1.3%), with *bla*_KPC-3_ being the most common (11/14, 78.6%), followed by *bla*_KPC-2_ (3/14, 21.4%).

There were some clear trends between the *bla*_NDM_ allele and genus (Fig. 1b). The *bla*_NDM-5_ gene was more likely to be found in *E. coli* than *Klebsiella* spp., followed by *Citrobacter* spp., and then *Enterobacter* spp. (90.0% vs 31.0% vs 15.0% vs 1.8%, respectively, *P*<0.008). The *bla*_NDM-1_ gene was more likely to be found in *Enterobacter* spp., followed by *Citrobacter* spp. and *Klebsiella* spp., and then *E. coli* (91.0% vs 64.0% vs 62.0% vs 6.5%, respectively, *P*<0.001). Finally, *bla*_NDM-7_ was more likely to be found in *Citrobacter* spp. than *E. coli*, *Enterobacter* spp. and *Klebsiella* spp. (19.0% vs 7.7% vs 3.5% vs 4.6%, respectively, *P*<0.005).

Organisms carrying *bla*_NDM-1_ were more likely to also encode resistance genes for other beta-lactamases, colistin, quinolones, phenicols, rifampicin and fosfomycin compared to organisms carrying *bla*_NDM-5_ (99% vs 95%, 4.6% vs 0.7%, 76.0% vs 31.0%, 40.0% vs 7.7%, 39.0% vs 9.2%, respectively, *P*<0.001). Organisms carrying *bla*_NDM-5_ were more likely to encode resistance genes for trimethoprim, sulphonamides, tetracyclines and macrolides compared to organisms carrying *bla*_NDM-1_ (94.0% vs 83.0%, 96.0% vs 92.0%, 67.0% vs 45.0%, 81.0% vs 59.0%, respectively, *P*<0.001).

### Clustering *bla*_NDM_-encoding plasmids

**Fig. 3.**
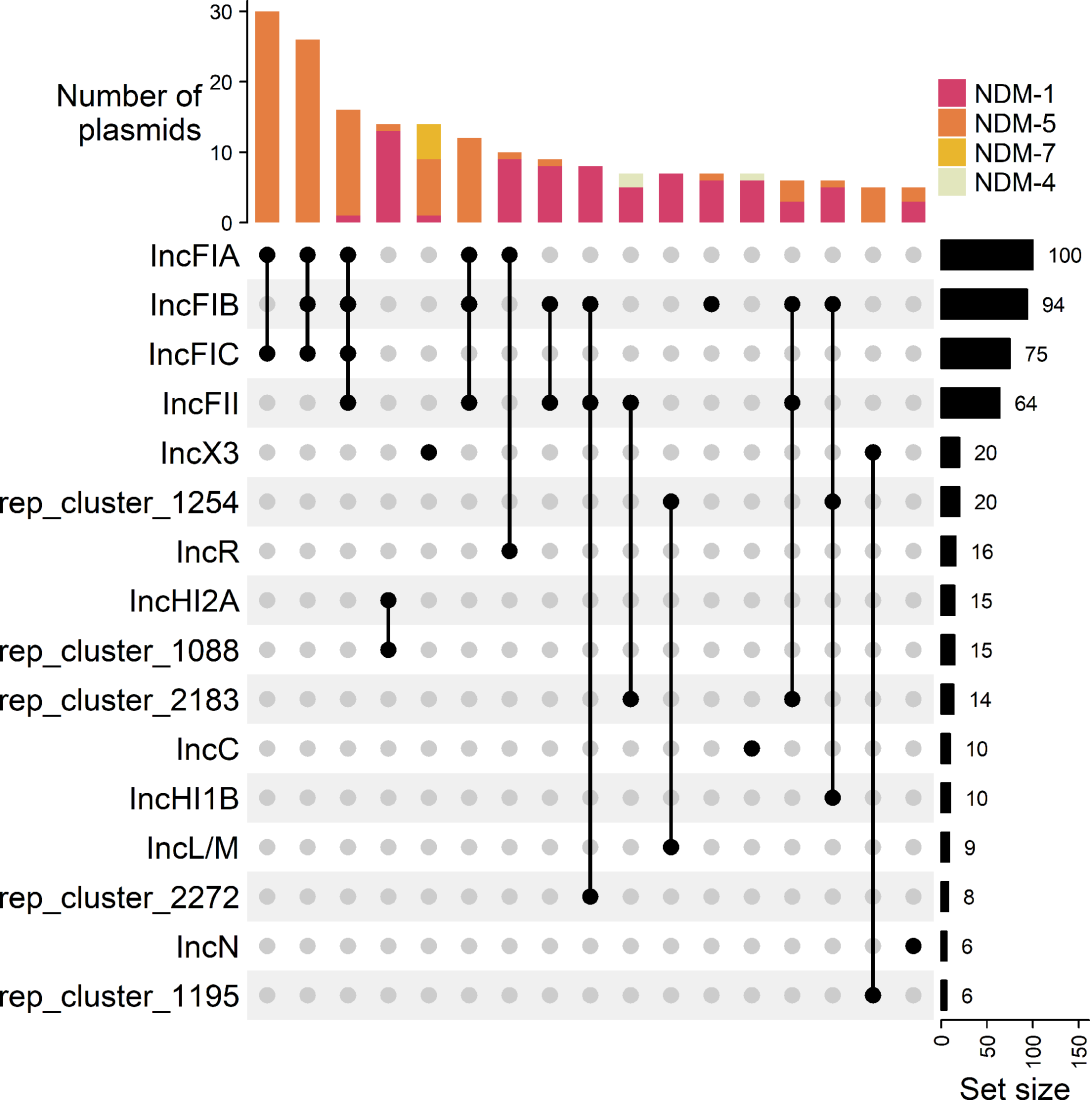
Top replicon combinations in complete *bla*_NDM_-encoding plasmids (*n*=226). Only replicon types present in six or more isolates and replicon combinations present in five or more isolates are plotted here. ‘Set size’ indicates the total number of replicons.

Using a hybrid sequencing approach on a subset of 237 isolates, we obtained 226 complete *bla*_NDM_-encoding plasmids (Table S2). Overall, plasmids ranged from 34.3 to 441.4 kb in size. There were 26 replicon types detected, with the most common types being IncFIA (106/226, 46.9%), IncFIB (98/226, 43.4%), IncFIC (79/226, 35.0%), IncFII (67/226, 29.6%) and IncX3 (20/226, 8.8%); however, 80.1% (181/226) of isolates had two or more different replicon types on the same plasmid (Fig. 3). The most common combinations of replicon types were IncFIA/FIC (30/226, 13.3%), IncFIA/IncFIB/IncFIC (26/226, 11.5%) and IncFIA/IncFIB/IncFIC/IncFII (16/226, 7.1%). Most plasmids were predicted to be conjugative (171/226, 75.7%), with a few predicted to be mobilizable (34/226, 15.0%) or non-mobilizable (21/226, 9.3%). There were some clear patterns between replicon type and *bla*_NDM_ type; *bla*_NDM-5_ was primarily found on IncF-type replicons (97/120, 80.8%), and *bla*_NDM-7_ was primarily found on IncX3 replicons (5/6, 83.3%), whereas *bla*_NDM-1_ was found on a variety of replicon types, including IncFIB, IncFII, IncR and IncHI2A. The *bla*_NDM_ plasmids encoded a mean number of 10 resistance genes, including *bla*_NDM_, and a mean number of 2.5 replicon types per plasmid. Most were assigned PTU-FE (60/226, 26.5%), PTU-X3 (19/226, 8.4%), PTU-HI2 (14/226, 6.0%) and PTU-E46 (11/226, 4.9%) or did not have a PTU assigned (59/226, 26.1%).

We attempted to cluster the complete *bla*_NDM_-encoding plasmids (*n*=226) from our dataset with the global collection of plasmids in the PLSDB database (*n*=59,895) [83] using MOB-suite [64,65]. The *bla*_NDM_ plasmids grouped into 64 primary clusters and 95 secondary clusters alongside PLSDB plasmids. The top five most populated primary clusters represented 38.4% of *bla*_NDM_ plasmids in our dataset (87/226), and the top ten most populated primary clusters represented 55.8% (126/226). This is in contrast to our previous data, where 86.6% of *bla*_KPC_ plasmids grouped in the top ten clusters and 78.6% of *bla*_OXA-48-type_ plasmids grouped in the top four clusters in Lerminiaux *et al*. [85,86], indicating there is high diversity among *bla*_NDM_ plasmids. No new primary or secondary clusters were observed in our Canadian dataset, indicating that all plasmids observed here are related to plasmids in PLSDB.

We examined the secondary clusters for evidence of horizontal plasmid transmission by searching for the same plasmid across different species and hospital sites. However, most secondary clusters had a clonal association, where the same secondary plasmid clusters were observed in isolates with the same ST found at different hospital sites in different years (Table S2). For example, one secondary cluster contained six *E. hormaechei* ST136 isolates with IncFIA/IncR plasmids encoding *bla*_NDM-1_ from three different sites in 2022 and 2023. Most secondary clusters had fewer than eight plasmids, indicating they represented only a small proportion of our dataset and that no major *bla*_NDM_ plasmid lineages have risen to dominance in the Canadian plasmid population. We summarized the main features of the top secondary clusters (Table 1), but overall, these represent a minor proportion of our dataset (65/226, 28.8%). The secondary clusters summarized here show that isolates were separated across multiple sites and across multiple years, which indicates these incidences likely are not closely related. The lack of trends observed in secondary plasmid clusters suggests that horizontal plasmid transmission is not playing a major role in *bla*_NDM_ spread.

**Table 1.** Summary features of top Canadian *bla*_NDM_-encoding plasmid secondary clusters among PLSDB

Secondary plasmid cluster	Number of PLSDB plasmids	Number of Canadian plasmids	Number of Canadian sites	Number of years	Number of species	Replicon∗	Mobility∗,†	*bla*_NDM_ allele	Average number of ARGs	Median size (kb)	Average DCJ-Indel distance‡	PTU∗,§
AQ043	413	14	7	7	7	IncX3	Conjugative	1, 5, 7	1	46.2	1.5	PTU-X3
AR038	5	8	5	5	1	IncFIA/FIA/FIC	Conjugative	5	8	120.4	4.3	PTU-FE
AR046	10	8	6	4	1	IncFIA/FIB/FIC/FII	Conjugative	5	11	140.4	2.0	PTU-?
AQ290	89	15	13	9	2	IncFIA/FIC	Conjugative	5	8	96.6	1.5	PTU-FE
AQ737	98	11	6	4	4	IncHI2A/rep_cluster_1088	Conjugative	1	14	289.3	3.8	PTU-HI2
AQ935	11	9	7	5	1	IncFIA/FIB/FII/FII	Conjugative	5	11	157.2	4.5	PTU-FE

∗Values indicate the most common genotype in the cluster and may not apply to all plasmids in the cluster.

†Values obtained from MOB-suite. Mobility is assigned based on presence of relaxase (mobilizable) and/or MPF proteins (conjugative) or absence of both (non-mobilizable).

‡DCJ-Indel distances calculated with pling.

§PTU values obtained from COPLA.

In addition to the clusters defined by MOB-suite, we clustered all *bla*_NDM_-encoding plasmids (*n*=226) using pling, which assigns plasmids to communities based on containment and rearrangement distances (specifically the DCJ-Indel model) [84]. Unlike MOB-suite, which clusters plasmids based on Mash distance to an existing plasmid database, pling groups plasmids based on the number of structural changes between plasmid pairs. Using the default containment threshold of 0.5 and DCJ-Indel threshold of 4, pling grouped our plasmids into 112 subcommunities, with an average subcommunity size of 2.02 plasmids, which, similar to MOB-suite, highlights the diversity of the *bla*_NDM_ plasmid dataset. There were five subcommunities with eight or more members, which correspond to the MOB-suite secondary clusters described in Table 1. Visualization of the plasmid network and subcommunities identified by pling is found in Fig. S1.

### Plasmid cluster prediction for incomplete *bla*_NDM_-encoding contigs

We attempted to reconstruct plasmids from isolates with incomplete *bla*_NDM_-encoding contigs using MOB-recon. However, during validation wherein we ran MOB-recon on a subset of Illumina-only assemblies for isolates from 2022 to 2023 for which we had complete hybrid-assembled plasmids (*n*=82), only 28.0% (23/82) of *bla*_NDM_-encoding contigs were predicted to be part of the same secondary cluster as their complete plasmid from their hybrid assembly. We do not believe this reflects the accuracy of MOB-suite, as we have shown in other work that it has accurately predicted secondary clusters for other carbapenemase-encoding contigs [85], but rather indicates the difficulty of assembling contiguous *bla*_NDM_-encoding plasmids (primarily IncF-type backbones as observed in Fig. 3) with short-read data alone. Conversely, this pattern was not observed in all plasmid types; for example, we noticed an association between *bla*_NDM-7_ and the IncX3 backbone (Fig. 3). A total of 52 (52/58, 89.7%) *bla*_NDM-7_-carrying isolates in our dataset encoded *bla*_NDM-7_ on an incomplete contig; almost all of these isolates (49/52, 94.2%) encoded an IncX3 replicon, and the majority (41/52, 78.8%) were predicted by MOB-suite to carry *bla*_NDM-7_ on the IncX3 backbone in the same secondary cluster. Likewise, 60/73 (82.2%) isolates with *bla*_NDM-5_ on an incomplete contig that also encoded an IncX3 replicon were predicted to encode *bla*_NDM-5_ in the same IncX3 secondary cluster. To examine if there was an elevated number of insertion sequence elements (IS, which generally encode a transposase) found on *bla*_NDM_-encoding plasmids with IncF-type replicons compared to *bla*_NDM_-encoding plasmids with other replicon types, which may contribute to difficulties in assembly and cluster prediction, we used ISEscan to count the number of insertion sequences on both groups of plasmids. There were significantly more IS elements identified on IncF-type *bla*_NDM_ plasmids (mean number of elements per kb: 0.135) compared to other replicon types (mean number of elements per kb: 0.115) (*W*=7,469, *P*<0.001), which may contribute to difficulty assembling complete IncF-type replicons. In addition, the IncX3 plasmids encoded an average of 1 ARG, whereas IncF-type plasmids had an average of 8 to 11 ARGs (Table 1), which are frequently associated with mobile elements [87]. Given that *bla*_NDM_-encoding contigs cannot accurately be assigned to the correct plasmid cluster, specifically in IncF-type plasmids, which represented most Canadian *bla*_NDM_ plasmids, we did not further assess *bla*_NDM_ plasmid backbones in incomplete or short-read-only assemblies.

### Occurrences of *bla*_NDM-5_ over multiple years at two hospital sites (A and B)

We examined *bla*_NDM-5_ occurrences at two sites that had the highest rates of *bla*_NDM_ and had been submitting data for multiple years to see if there was evidence of horizontal transmission among single sites [A (2016–2023, *n*=44) and B (2013–2023, *n*=55)]. There were several complete plasmids that grouped in the same primary clusters but different secondary clusters and were quite distinct from each other (based on Mash distance, replicon types, size and ARG profile). We analysed the set of *bla*_NDM-5_ plasmids at each site using pling. For both sites, we found no evidence of horizontal gene transfer between isolates. Pling calculates the minimum number of DCJ, insertion or deletion events between all plasmid pairs, and we found that all DCJ-Indel distances for site A were >13, with one pair as an exception with a DCJ-Indel distance of 4. Likewise, all DCJ-Indel distances between the plasmids from site B were >14, indicating these plasmids are not recently related. It is likely that horizontal gene transfer has occurred at these sites, but occurred at a scale that we cannot detect using these tools or that multiple recombination and gene gain/loss events have occurred since transfer happened. Alternatively, this data do not discount that these sites observe multiple new acquisitions of *bla*_NDM-5_ over the course of the sampling.

### *bla*_NDM-5_ plasmid evolution in *E. coli* ST167 over 1 year at hospital site C

**Fig. 4.**
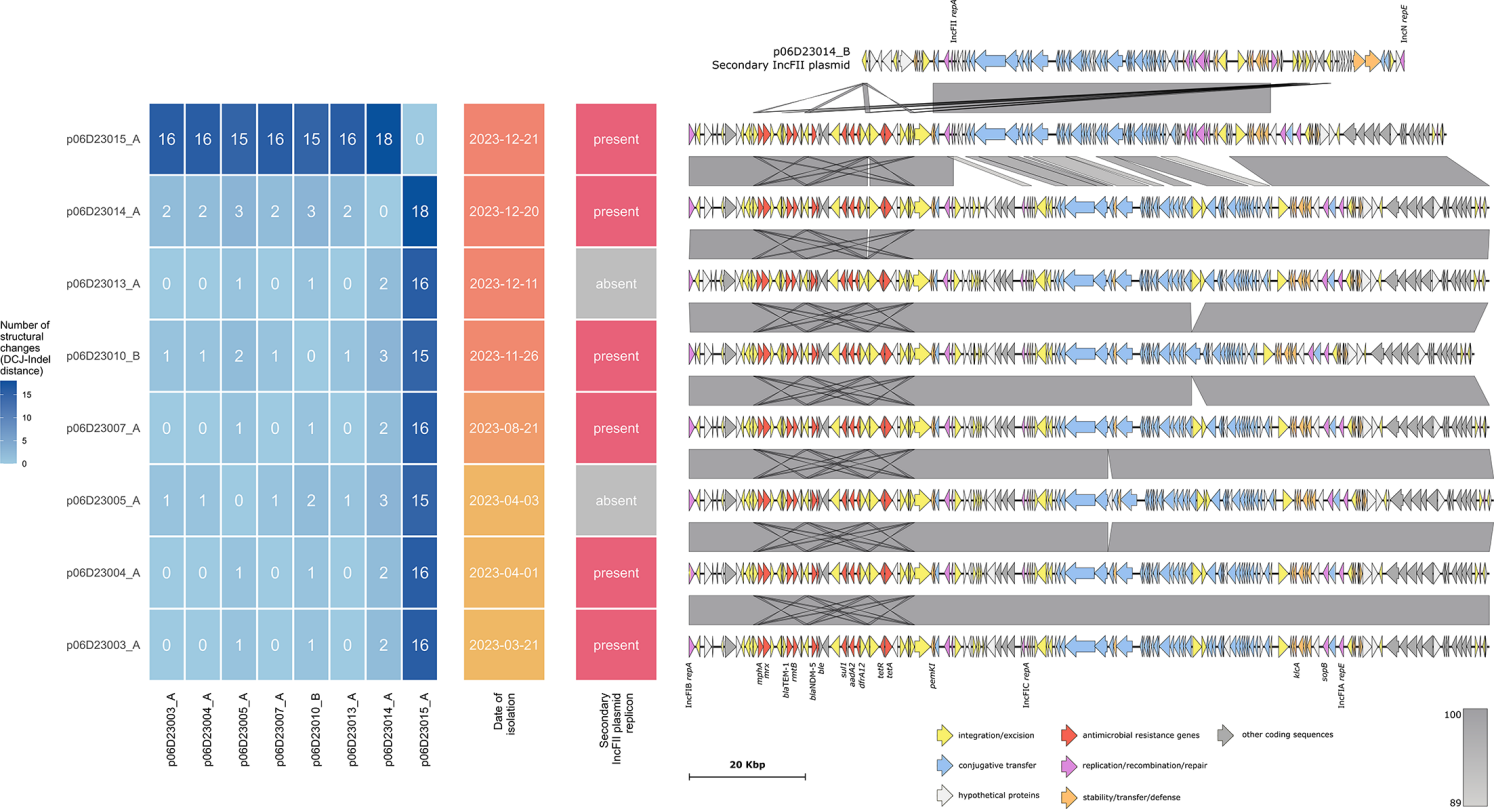
*bla*_NDM_-encoding plasmids from eight *E. coli* ST167 isolated from a single hospital site over 1 year. Heatmap values indicate DCJ-Indel distances obtained with pling; lower numbers indicate fewer structural changes. Presence of a second IncFII plasmid in these isolates is indicated by present/absent. Plasmid structures are indicated on the right, and grey shading indicates percent identity obtained with blastn. Coding sequences are coloured by function, and not all coding sequences are labelled. The secondary IncFII plasmid from isolate 06D23CPO014 (p06D23014_B), which does not encode *bla*_NDM_, is included at the top of the gene plot for comparison with the hybrid structure of p06D23015_A.

To examine plasmid structural changes over a shorter time scale, we examined eight cases of *E. coli* ST167 encoding *bla*_NDM-5_ on IncF-type plasmids from hospital site C isolated across 9 months in a single year (2023). Historically, this site had a single *bla*_NDM_-positive isolate each year from 2019 to 2022 and two additional *K. pneumoniae* isolates in 2023 that appeared unrelated. There were limitations to this example, as we do not know the sampling strategy for every site, and isolates may have been missed due to screening practice limitations. We conducted ONT sequencing on these eight cases from 2023 to obtain the complete *bla*_NDM_ plasmid sequences. All but one plasmid was 137 kb in size, had IncFIA/FIB/FIC replicons and varied between 0 and 3 DCJ-Indel distances among themselves using pling (Fig. 4), which indicates that they are closely related. Five of these seven were co-harboured an additional 93 kb IncFII plasmid. The eighth *bla*_NDM_-encoding plasmid (p06D23015) was isolated most recently 1 day following the previous case, was 130 kb in size, had IncFIA/FIB/FII replicons, had DCJ-Indel distances of 15 to 18 from the remaining plasmids and was placed in a different secondary cluster by MOB-suite. This plasmid shared sequence with both the IncFIA/FIB/FIC *bla*_NDM_-encoding plasmid from the first group as well as the second IncFII plasmid present in most isolates, indicating a recombination event likely occurred between these two plasmids in the eighth isolate clone to produce a hybrid plasmid encoding both *bla*_NDM_ and IncFIA/FIB/FII replicons.

This example illustrates two contrasting ideas: first, how a plasmid can maintain its structure and genomic organization in a clonal lineage over 9 months of time, indicating that this plasmid is relatively stable. Second, we have found an example of how a single genetic event (recombination with a co-resident plasmid) can dramatically change plasmid structure and consequently classification (different MOB-suite cluster, high number of genetic change events predicted by pling), making it incredibly difficult to track *bla*_NDM_ plasmids across the country for surveillance purposes.

### Isolates encoding both *bla*_NDM_ and *bla*_OXA-48-type_ carbapenemases

We observed 11.4% (118/1,032) of isolates encoding both *bla*_NDM_ and *bla*_OXA-48-type_ carbapenemases across 36 hospital sites. Isolates encoding both *bla*_OXA-48-type_ and *bla*_NDM_ represented 27 STs found in *K. pneumoniae* (70/118, 59.3%), *E. coli* (46/118, 39.0%) and *Providencia* spp. (2/118, 0.3%). The most common STs were *E. coli* ST147 (16/118, 13.6%), *K. pneumoniae* ST16 (12/118, 12.2%), *E. coli* ST2581 (9/118, 7.6%), *E. coli* ST410 (9/118, 7.6%), *K. pneumoniae* ST231 (8/118, 6.8%) and *K. pneumoniae* ST395 (8/118, 6.8%). *E. coli* ST147 represented 34.7% (16/46) of *E. coli* encoding both *bla*_NDM_ and *bla*_OXA-48-type_. Clonal transmission appears to have occurred at a single site in 2023 with four *E. coli* ST2851 isolates (*bla*_NDM-5_ and *bla*_OXA-181_, 0–6 single nucleotide variants (SNVs) using 93.7% of the genome). Eight *K. pneumoniae* ST231 isolates (*bla*_NDM-5_ and *bla*_OXA-232_, 26–130 SNVs using 93.2% of the genome) were observed over 6 years across six different sites and had plasmids grouped in the same secondary cluster, possibly representing clonal association with these plasmids. All other isolates with the same STs did not appear to carry the same plasmid combinations. Overall, this indicates that there was diversity in *bla*_NDM_ and *bla*_OXA-48-type_ plasmid clusters among STs, suggesting multiple introductions of co-occurring *bla*_NDM_ and *bla*_OXA-48-type_ plasmids.

**Fig. 5.**
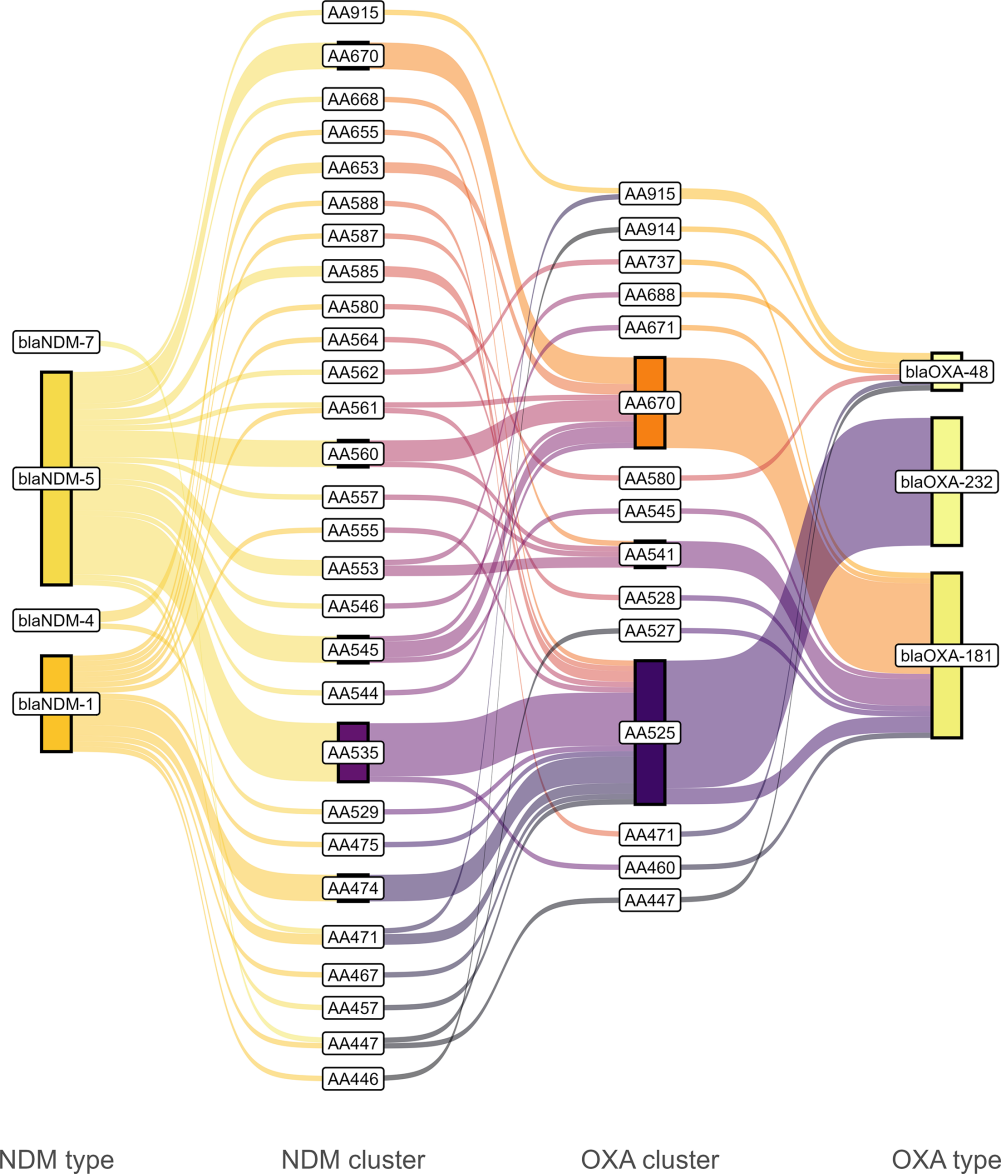
Relatedness among *bla*_NDM_ type, *bla*_NDM_ plasmid cluster, *bla*_OXA-48-type_ plasmid cluster and *bla*_OXA-48-type_ allele for 62 isolates that encoded NDM and *bla*_OXA-48-type_ genes on complete and closed replicons. Bar height indicates number of isolates, and colours are assigned alphabetically and are meaningless. Cluster numbers corresponding to custom MOB-suite secondary clusters (Mash distance cutoff <0.025). Identical cluster codes that are connected between ‘NDM_cluster’ and ‘OXA_cluster’ indicate plasmids where *bla*_NDM_ and *bla*_OXA-48-type_ genes are encoded on the same contig (*n*=9).

About half the isolates (62/118, 52.5%) had complete *bla*_NDM_ and *bla*_OXA-48-type_ plasmids, and four additional isolates had either *bla*_OXA-48-type_ (*n*=3) or both *bla*_NDM_ and *bla*_OXA-48-type_ (*n*=1) on the chromosome. We plotted the relationship among *bla*_NDM_ type, *bla*_OXA-48-type_ type and their corresponding plasmid secondary clusters for complete *bla*_OXA-48-type_ and *bla*_NDM_ plasmids in Fig. 5. There was more diversity in *bla*_NDM_ plasmids than in *bla*_OXA-48-type_ plasmids (30 secondary clusters vs 16 secondary clusters for *bla*_OXA-48-type_ plasmids). Of all isolates co-harbouring *bla*_NDM_ and *bla*_OXA-48-type_, 67.7% (42/62) fell into 1 of 2 *bla*_OXA-48-type_ clusters that have been previously observed in Canada [86] (equivalent to secondary clusters AB484 and AA836, respectively); the first *bla*_OXA-48-type_ cluster being AA525 (*n*=27, 6.1 kb rep_cluster_1195/ColKP3), which almost exclusively represented *bla*_OXA-232_ and was found associated with multiple types of *bla*_NDM_ plasmids, including AA535 (95 kb IncFIA/IncFIC in *K. pneumoniae*). The second *bla*_OXA-48-type_ cluster, AA670 (*n*=17, 51.5 kb IncX3), was exclusively associated with *bla*_OXA-181_ and often found alongside *bla*_NDM_ clusters AA545 (115 kb IncFIA/IncFIA/IncFIC in *E. coli*) and AA560 (160 kb IncFIA/FIB/FII/FII in *E. coli*, equivalent to AQ935 in Table 1).

**Fig. 6.**
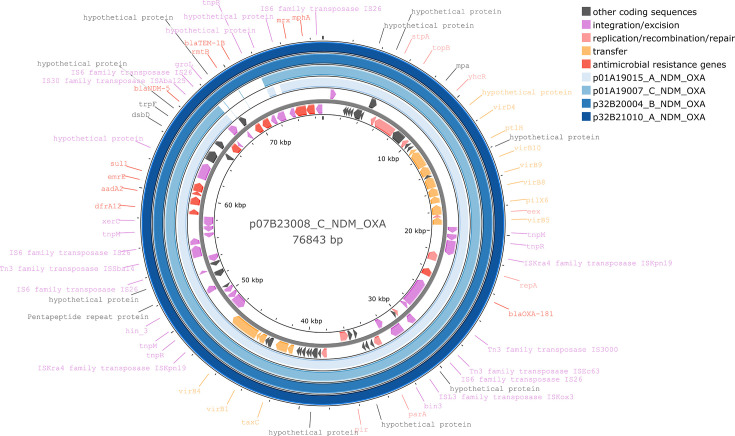
Plasmid map of p07B23008_C_NDM_OXA plasmid (76.8 kb). This plasmid has an IncX3 backbone and encodes both *bla*_NDM-5_ and *bla*_OXA-181_ in *K. pneumoniae* ST16. Four other plasmids encoding both *bla*_NDM-5_ and *bla*_OXA-181_ from other *K. pneumoniae* ST16 isolates in this study were aligned by blast at >99% identity (outer rings). Coding sequences are coloured by function, and not all coding sequences are labelled.

We observed nine plasmids that encoded both *bla*_NDM_ and *bla*_OXA-48-type_ carbapenemases. Of these, five were found in *K. pneumoniae* ST16 isolated from three sites in two provinces from 2019 to 2023, which encoded *bla*_NDM-5_ and *bla*_OXA-181_ on IncX3/rep_cluster_1195 replicons (Fig. 6). The plasmids were 70–76 kb, grouped in the same secondary cluster, and were predicted to be conjugative by MOB-suite. In addition to carbapenemase genes, they also encoded *aadA2*, *dfrA12*, *mph(A)*, *qnrS1* and *sul1* resistance genes. Pling grouped all plasmids in the same subcommunity, and the DCJ-Indel distances varied from 1 to 5, indicating there are some structural differences despite having >99.0% pairwise identity. The other four plasmids were found at unique sites and species/ST combinations and did not cluster together. They were an 89 kb IncFIA/rep_cluster_1418 mobilizable plasmid in *K. pneumoniae* ST11 encoding *bla*_NDM-1_ and *bla*_OXA-48_, a 106 kb IncL/M conjugative plasmid in *K. pneumoniae* ST383 encoding *bla*_NDM-5_ and *bla*_OXA-48_, a 132 kb IncFIA/FIC/rep_cluster_1195 conjugative plasmid in *E. coli* ST361 encoding *bla*_NDM-5_ and *bla*_OXA-181_ and a 233 kb IncC conjugative plasmid in *Proteus mirabilis* ST146 encoding *bla*_NDM-1_ and *bla*_OXA-48_.

### Genetic context of *bla*_NDM_ genes

**Fig. 7.**
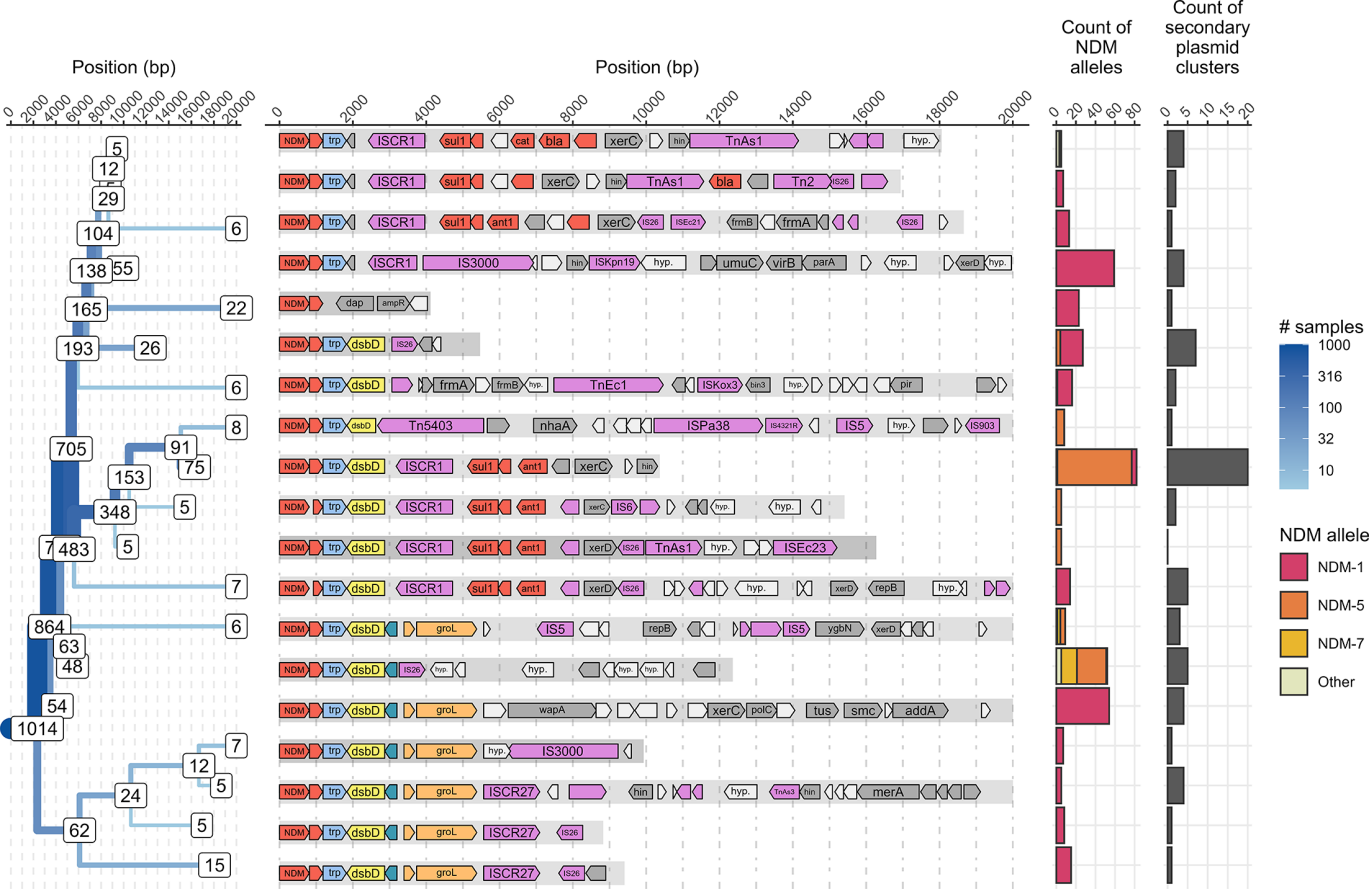
Splitting tree for most common (≥5 contigs) structural variants downstream of *bla*_NDM_ (*n*=1,024). Labels on branches with <5 contigs are not shown. Contigs with two *bla*_NDM_ genes on the same replicon (*n*=5) were excluded, as were contigs with truncated *bla*_NDM_ genes (*n*=3). Genome annotations by Prokka are shown in the middle plot and not all genes are labelled. Genes are coloured by function: red indicates ARGs, blue indicates *trpF*, yellow indicates *dsbD*, aqua indicates *cutA*, orange indicates *groESL*, pink indicates mobile elements, grey indicates coding sequences of other functions and white indicates hypothetical proteins. Homologous regions are indicated by bar shading behind genes. The distribution of *bla*_NDM_ alleles and number of secondary plasmid clusters where that structural variant is found in complete plasmids are shown on the right.

We investigated the region flanking the *bla*_NDM_ genes. The Tn*125* transposon has been proposed as the ancestral transposon responsible for mobilizing *bla*_NDM_ [31], and all *bla*_NDM_-encoding isolates to date harbour a complete or fragmented IS*Aba125*, an IS constituting Tn*125*, immediately upstream of *bla*_NDM_, which contains a promoter region [8,13]. We followed the protocol described by Acman *et al*. [13] to investigate the downstream flanking region of all *bla*_NDM_ contigs encoding a single, complete *bla*_NDM_ gene (*n*=1,024). The upstream homology of *bla*_NDM_ falls quickly as observed previously [13,32], and we observed many of the same downstream variants as Acman *et al*. [13] (Fig. 7). There were no clear patterns between structural variant and plasmid type. The largest observation of a single structural variant represented 8.0% of isolates (82/1,024), most of which encoded *bla*_NDM-5_ (93.9%, 77/82), but there was no association between site, year or organism. This same structural variant was observed in 24 different secondary plasmid clusters identified by MOB-suite, further supporting the idea proposed by Acman *et al*. [13] that transposons have played a larger role in *bla*_NDM_ spread than plasmid mobility.

## DISCUSSION

We examined the prevalence and distribution of *bla*_NDM_-producing *Enterobacterales* and their plasmids in Canada from 2010 to 2023. This is one of the larger *bla*_NDM_-encoding plasmid investigations to date. Our results show that *bla*_NDM_ plasmid diversity is extremely high and that unlike *bla*_KPC_ and *bla*_OXA-48-type_, which have conserved plasmid trends in Canada over the same period [85,86], conserved plasmid transfer has not proven to play a major role in *bla*_NDM_ dissemination across Canada. We clustered 226 complete *bla*_NDM_-encoding plasmids, which represented ~21.9% of our total *bla*_NDM_ dataset, and found a lack of dominant clusters, where secondary clusters each encoded a small proportion (<6.6%) of total plasmids. While we occasionally saw clonal links within secondary clusters, these were often represented by fewer than six isolates. Additionally, the most common STs all had a variety of plasmid types. We searched for patterns of plasmid transmission at two hospital sites with the highest numbers of *bla*_NDM_ cases over 8 years and did not find evidence of horizontal transmission within these sites using the most current bioinformatic tools available for plasmid clustering. Given the increasing prevalence of *bla*_NDM_-producing *Enterobacterales* over the last 5 years in Canada [12] and worldwide [7,18, 22], our findings contribute to a better understanding of the dissemination of *bla*_NDM_-producing *Enterobacterales* and their plasmids.

Two recent studies of *bla*_NDM_ plasmids may provide insight into the high plasmid diversity and lack of horizontal transfer across Canada. First, Acman *et al*. [13] argued that global dissemination of *bla*_NDM_ was primarily driven by successive between-plasmid transposon jumps within hosts, and subsequent plasmid exchange was much more restricted. They noted that if a plasmid transfers to a new host but fails to establish itself, that brief period of co-residency can provide opportunities for between-plasmid transposon jumps. Our data may support this idea of transposon-mediated *bla*_NDM_ spread, as we found a lack of evidence of widespread plasmid transfer and no relationship between plasmid backbone and transposon structural variant (Fig. 7), which may be a consequence of the inability of *bla*_NDM_ plasmids to establish themselves in new bacterial hosts. Currently, we lack effective tools to confirm these transposon jumps in large-scale datasets. In addition, we could not capture isolates that encoded *bla*_NDM_ and then lost their plasmid or transposon in this surveillance-based study, and these transient events are likely contributing to *bla*_NDM_ transmission and dissemination globally. Second, David *et al*. [16], who noted that *bla*_NDM_ is found in multiple plasmid backgrounds and multiple lineages of *K. pneumoniae*, proposed that high rates of recombination and rearrangement among plasmids could partly explain both the transient association between lineages and plasmids as well as the absence of any single dominant plasmid found across multiple lineages. We highlighted one example of plasmid recombination, which resulted in a plasmid from *E. coli* clonal lineage ST167 being grouped into a different secondary cluster over a sample period of 9 months (Fig. 4), but also showed relative plasmid stability in this lineage, indicating the randomness of recombination and rearrangement events. We cannot comment on the frequency of these events; fine-scale datasets are needed to reveal the extent and frequency of structural changes.

A recent study showed that plasmids can be maintained in *E. coli* lineages for centuries [88], which suggests that certain strains are well adapted to their plasmids [89]. These strains can then potentially act as sources for transposon or plasmid movement to other less suitable isolates where those mobile elements may be less likely to persist [90], as described in the source–sink solution to the plasmid paradox [91]. Most secondary plasmid clusters observed here contained isolates from multiple years that often had a clonal link, and these isolates may serve as hubs or sources that can disseminate *bla*_NDM_-encoding plasmids or transposons to other less proficient strains.

The *bla*_NDM_ and *bla*_OXA-48-type_ co-carriage rates we observed here were ten times higher than those observed in the USA [92], although they reported that the number of carbapenemase-producing *Enterobacterales* isolates carrying more than one type of carbapenemase had increased from 1.1% (*n*=54) in 2018 to 3.2% (*n*=223) in 2022. The *bla*_NDM-5_ and *bla*_OXA-232_ co-carriage in *K. pneumoniae* ST231 has not been reported on previously, although other *K. pneumoniae* isolates have been found to carry similar plasmids [93,94], and may indicate an emerging trend.

The co-localization of *bla*_NDM_ and *bla*_OXA-48-type_ carbapenemases on the same plasmid backbone was only recently reported [95]. Zuo *et al*. [95] described a 63.2 kb IncX3 plasmid encoding *bla*_NDM-5_ and *bla*_OXA-181_ in an *E. coli* isolate from Japan (AP028870.1), but this structure differs from the ones we described here. The co-carriage of *bla*_NDM_ and *bla*_OXA-58-type_ on the same plasmid has been observed in *Acinetobacter* [96,97]. We identified nine cases of *bla*_NDM_ and *bla*_OXA-48-type_ co-localized on the same plasmids, five of which were from *K. pneumoniae* ST16 and harboured *bla*_NDM-5_ and *bla*_OXA-181_, which did not appear to be directly linked epidemiologically or genetically. Hendrickx *et al*. [98] identified a plasmid containing both *bla*_NDM_ and *bla*_OXA-48-type_ that grouped in the same AQ051 cluster (NZ_CP068835.1), which was also isolated from *K. pneumoniae* ST16. *K. pneumoniae* ST16 is a global multidrug-resistant clone that has been previously documented to carry both *bla*_OXA-48-type_ and *bla*_NDM_ carbapenemases on separate plasmids [99,100]. Given the predicted conjugative ability and co-localization of many other ARGs on these plasmids, the co-localization of two types of carbapenemases is concerning and should be monitored for future spread.

Although *bla*_NDM_ gene carriage was associated with a variety of organisms, the most common were *E. coli* and *K. pneumoniae* spp. complex. In the UK from 2014 to 2016, *K. pneumoniae* was the dominant *bla*_NDM_-encoding organism, which primarily encoded *bla*_NDM-1_, and *E. coli* encoded a mix of *bla*_NDM-1_ and *bla*_NDM-5_ [21]. Our dataset shows a shift in more recent years (2018–2023), where most *bla*_NDM_-encoding isolates in Canada were *E. coli* encoding mainly *bla*_NDM-5_. Others have also observed this rise of *bla*_NDM-5_ across the globe, with *bla*_NDM-5_ cases becoming more prevalent than *bla*_NDM-1_ since 2017 [7,13, 22, 101]. The dominant STs carrying *bla*_NDM-1_ observed here were also found in other parts of the world, all of which are known high-risk clones. *E. coli* ST167, ST405 and ST648 are commonly found carrying *bla*_NDM_ in Europe [18,19], the United Kingdom [21] and in global datasets [7,26]. Fewer *E. coli* ST131 and ST410 were observed in Canada compared to other regions [7,22]. As in Canada, *bla*_NDM_-encoding *K. pneumoniae* ST147 and ST11 were common in Europe [16], Singapore [15] and the United Kingdom [17,21]. Likewise, *E. hormaechei* subsp*. xiangfangensis* ST171 was common in Canada and elsewhere in North America and Asia [102].

Most of the plasmids observed in our study had IncF-type replicons, which is not a popular *bla*_NDM_ backbone in other studies. IncX3 has been identified as the most common *bla*_NDM_ plasmid backbone globally [8,26] and in China [22,103]. Similarly, studies describing *bla*_NDM_ outbreaks have also focused on IncX3 as the dominant backbone [27,28]. A recent study [104] showed that *bla*_NDM-5_ likely mobilized to an IncX3 plasmid, and subsequent IncX3 plasmids have acquired mutations in *bla*_NDM_ to produce different alleles that have spread globally; however, this plasmid type is not yet dominant in Canada. *K. pneumoniae* frequently harbours *bla*_NDM_ on IncF-type backbones across a global collection [105] and in Europe [16], and we observed most IncF backbones in *E. coli*. IncF-type plasmids are large, mosaic, low copy number conjugative, have a narrow host range, and often encode multiple ARGs [88,106,108]. They are more likely to encode multiple replicons compared to other plasmids [109,110], which we observed here. IncF plasmids can rapidly gain and lose genes in shorter sampling periods (~3 years) compared to other plasmids such as Col-type, which can remain the same length over longer sampling periods (~10 years) [111]. The IncF plasmids here encoded high numbers of other ARGs and mobile elements, which facilitate genetic exchange [87,112], further supporting the idea that rapid recombination makes tracking *bla*_NDM_ plasmid transfer events difficult due to frequent reshuffling of genes. The diversity of IncF plasmids in Canada could also be attributed to multiple independent introductions of *bla*_NDM_, as *bla*_NDM_ cases are significantly associated with international travel [12]. Large-scale sequencing of susceptible isolates with IncF-type plasmids may help resolve movement of transposons to new backbones, but given their diversity and rapid recombination rates, this would require a thorough sampling strategy and would be a challenging endeavour.

Determining plasmid relatedness is challenging due to backbone plasticity and frequent rearrangements [113]. We used a combination of MOB-suite [64,65] to cluster complete plasmids and pling [84] to investigate the structural differences within plasmid clusters. Pling is a new tool that examines plasmid relatedness while accounting for structural differences caused by transposons and other mobile elements frequently found on plasmids. While we did not conduct a thorough validation, pling and MOB-suite were generally in agreement for plasmid grouping and both revealed the diversity in our dataset. Pling’s DCJ-Indel distances are a measure of structural differences or evolutionary events between pairs of plasmids, which are not captured by MOB-suite’s clustering approach. We see an advantage to using pling beyond the surveillance level for tracking transmission in fine-scale outbreaks, as done with our example for *bla*_NDM-5_ plasmids in *E. coli* ST167 at hospital C.

The inability of MOB-recon to accurately predict clusters for *bla*_NDM_-encoding contigs in most of our short-read-only data made it difficult to infer plasmid backbone presence across our entire dataset. Short-read assemblers often struggle in regions with ARGs and insertion elements [25,114, 115], which makes assembling IncF-type backbones with their high numbers of insertion sequences and ARGs particularly challenging without long-read data. However, even if we were to have obtained long-read sequences for all plasmids in our dataset, the clusters would likely still be very diverse, given the diversity we observed in our closed plasmid dataset. For investigating *bla*_NDM_ plasmids in future studies, we would recommend still including long-read sequencing data for plasmid backbone and structure confirmation. Overall, we have observed that available tools used for plasmid clustering do not perform well on Canadian *bla*_NDM_ plasmids due to the nature of genetic diversity and instability of certain plasmid types (i.e. IncF-type plasmids). We have reiterated previously reported observations on the diversity of the *bla*_NDM_-harbouring transposon and concluded this was also not a viable target for clustering *bla*_NDM_ cases over long-term surveillance. Although we are still observing multiple introductions of *bla*_NDM_ via travel, this does not fully explain the diversity of cases, and our observations indicate a lack of clustering is due to multiple factors, including both genetic and epidemiological contributors. Knowledge of different plasmid backbones and their disposition to rapid genetic change is critical in making conclusions about the relatedness of cases when describing both outbreak and surveillance data.

## Supplementary material

10.1099/mgen.0.001415 Fig. S1.

10.1099/mgen.0.001415 Table S1.

10.1099/mgen.0.001415 Uncited Table S2.

## Abbreviations

ARGs: antimicrobial resistance genes
CNISP: Canadian Nosocomial Infection Surveillance Program
DCJ-Indel: Double Cut and Join Indel
IS: insertion sequence element
ONT: Oxford Nanopore Technologies
PTU: plasmid taxonomic unit
SNV: single nucleotide variant
STs: sequence types
