# Supplementary material for: Plasmid genomic epidemiology of blaNDM carbapenemase-producing Enterobacterales in Canada from 2010 to 2023
Source: Microb Genom. 2025 Aug 4;11(8):001415. doi: 10.1099/mgen.0.001415 (PMC12321488; doi:10.1099/mgen.0.001415)
Supplement: Fig. S1. [file mgen-11-01415-s001.pdf]

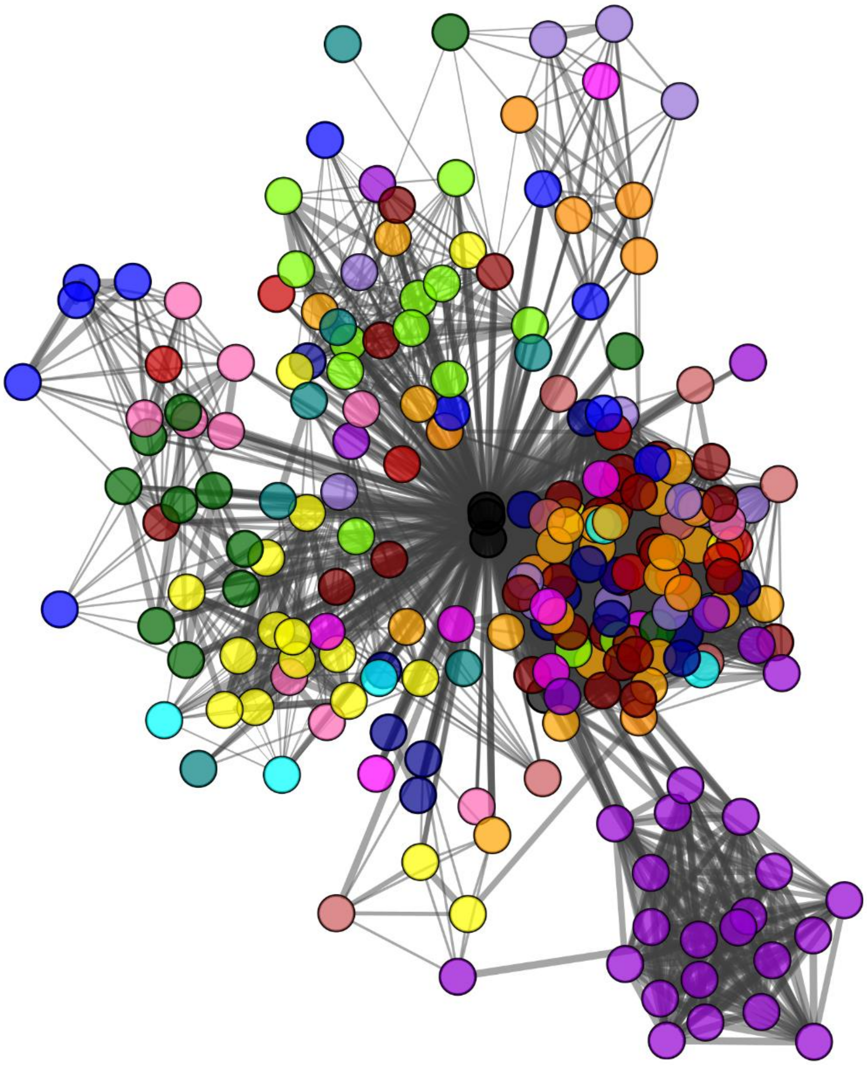

Supplementary Figure S1: Pling network of all bla_{NDM}-encoding plasmids (n=232). Pling was ran using the default contaminant threshold of 0.5 and DCJ-Indel threshold of 4. Nodes represent plasmids, colours represent different subcommunities with DCJ-Indel distances < 4 (n=112 subcommunities; note the same colours have been used for multiple plasmid subcommunities), and edges connect plasmids that have a containment distance < 0.5. Edge widths correspond to DCJ-Indel values, with wider widths corresponding to lower DCJ-Indel values (i.e. fewer structural changes). Edge lengths are meaningless.
